# Analysis of complete blood counts and derived inflammatory indices with non-suicidal self-injury and suicide attempt in adolescents with major depressive disorder

**DOI:** 10.3389/fpsyt.2026.1781733

**Published:** 2026-03-25

**Authors:** Jing Liu, Xiaoyan Zhai, Chenyang Gao, Shipan Zhang, Di Fei, Xueru Zhang, Man Jin, Yumei Wang

**Affiliations:** 1 Department of Psychiatry, Hebei Medical University, Shijiazhuang, Hebei, China; 2 Department of Clinical Psychology, Hebei General Hospital, Shijiazhuang, Hebei, China; 3 Department of Psychiatry, The First Hospital of Hebei Medical University, Shijiazhuang, Hebei, China; 4 Department of Psychology, Shandong Provincial Hospital Affiliated to Shandong First Medical University, Jinan, Shandong, China

**Keywords:** adolescent, inflammation, major depressive disorder (MDD), neutrophil-to-lymphocyte ratio (NLR), non-suicidal self-injury (NSSI), suicide attempt (SA)

## Abstract

**Background:**

Non-suicidal self-injury (NSSI) and suicide attempt (SA) are highly comorbid in adolescents with major depressive disorder (MDD), yet their distinct biological correlates are unclear. This study investigated the potential of complete blood count (CBC) parameters and derived inflammatory markers to differentiate clinical subtypes of adolescent MDD with NSSI and/or SA.

**Methods:**

A retrospective cross-sectional study was conducted involving 618 adolescent inpatients with MDD. The participants were categorized into four clinical subgroups: pure MDD group (without NSSI and SA, n=382), NSSI-only group (n=131), SA-only group (n=41), and NSSI+SA comorbid group (n=64). CBC parameters and derived inflammatory indices, including the neutrophil-to-lymphocyte ratio (NLR), monocyte-to-lymphocyte ratio (MLR), platelet-to-lymphocyte ratio (PLR), and systemic immune-inflammation index (SII), were compared across groups. Statistical analyses included Kruskal-Wallis H tests with *post-hoc* comparisons, multivariate logistic regression, and receiver operating characteristic (ROC) curve analysis.

**Results:**

The NSSI+SA comorbid group exhibited a robust inflammatory signature, with significantly elevated neutrophils, NLR, MLR, PLR, and SII, alongside reduced lymphocytes, compared to all the other subgroups (all *p* < 0.008). NLR was identified as a strong independent risk factor for the NSSI+SA comorbid state (odds ratios [ORs] ranging from 2.84 to 3.69). The NSSI-only group specifically exhibited elevated platelet crit (PCT) compared to the pure MDD group (*p* = 0.004). In contrast, the SA-only group showed no distinct inflammatory profile compared to the pure MDD or NSSI-only groups.

**Conclusion:**

NSSI and SA behaviors in adolescents with MDD are associated with distinct inflammatory biological bases. NSSI+SA comorbidity is linked to robust activation of the innate immune response, with elevated levels of NLR, MLR, PLR, and SII serving as potential auxiliary biomarkers for its identification.

## 1 Introduction

Major depressive disorder (MDD) is a severe mental illness that is not only a leading cause of health-related disability but also a major contributor to the global disease burden (1, 2). The first peak onset of depression occurs in early adolescence; with studies indicating that approximately 25% of adolescents experience a depressive episode by the age of 19 (3). The prevalence of MDD among adolescents has shown a significant increasing trend in recent years (4). Adolescent depression portends substantial long-term risks, including impaired social and occupational functioning, diminished quality of life, and a markedly elevated propensity for self-injurious and suicidal behaviors (5, 6).

Non-suicidal self-injury (NSSI) and suicide attempts (SA) are highly prevalent among youth and often co-occur, both of which predict later suicide (7–9). Globally, suicide ranks as the fourth leading cause of death among individuals aged 15–19 (10). Longitudinal evidence suggests that NSSI frequently occurs before SA, yet not everyone who engages in NSSI eventually attempts suicide (11), highlighting the need to identify factors that differentiate these behavioral trajectories A global meta-analysis reported that the lifetime and 12-month prevalence of NSSI among children and adolescents between 1989 and 2018 were 22.1% and 19.5%, respectively, while those of SA were 6.0% and 4.5%, respectively (12). Among adolescents with MDD, the reported prevalence of NSSI is notably higher, ranging from 51.0% to 81.9% (13–15), and the co-occurrence of NSSI with MDD substantially amplifies suicide risk (16). Collectively, MDD, NSSI, and SA represent a critical public health challenge for the adolescent population worldwide (17, 18). Therefore, it is crucial to accurately identify and differentiate MDD subtypes with co-occurring NSSI and/or SA to enable targeted interventions and reduce mortality.

However, the current assessment of NSSI and SA relies heavily on patients’ subjective reports and lack objective and quantifiable biological markers. This limitation impedes early identification and risk stratification. Inflammation may serve as a potential biological link between MDD, NSSI, and SA. A compelling body of evidence has established a link between depression and immune system dysregulation, with chronic low-grade inflammation emerging as a key pathophysiological mechanism in MDD (19, 20). Notably, studies have shown that elevated inflammatory levels are more closely associated with specific symptom clusters in depressed patients, such as impulsivity, aggression, and suicidal ideation (21–23). Given that this “behavioral activation/impulsivity” symptom cluster represents a core feature of both NSSI and SA (24, 25), it is plausible that inflammation may play a key role in linking MDD to the manifestation of NSSI and SA behaviors.

The complete blood count (CBC) is a ubiquitous, reproducible, and cost-effective hematological test. The derived indices, including the neutrophil-to-lymphocyte ratio (NLR), platelet-to-lymphocyte ratio (PLR), monocyte-to-lymphocyte ratio (MLR), and systemic immune-inflammation index (SII) are considered relatively stable and promising systemic inflammatory biomarkers (26), with NLR and PLR demonstrating particular predictive value in assessing systemic inflammatory status (27). These markers offer practical advantages for clinical translation due to their routine availability and low cost.

Previous studies have reported associations between such markers and depression or suicide (28, 29). For instance, elevated MLR has been found in MDD patients with a history of SA (30), while higher PLR and platelet count (PLT) are associated with greater depression severity and suicide risk (31, 32). However, research specifically linking inflammation to NSSI remains relatively limited and findings have been inconsistent Zheng et al. found elevated MLR and PLR in adolescents with mood disorders and NSSI, but no significant change in NLR (33). Another study showed that among individuals with NSSI and SA, only the white blood cell count (WBC) differed significantly, with no notable differences observed in PLT, NLR, MLR, PLR, or SII (34). These discrepancies may be attributable to clinical heterogeneity across samples, particularly the failure to distinguish between individuals with NSSI-only, SA-only, and comorbid conditions Furthermore, NLR and PLR have also been shown to differ significantly across sexes and age (35). Therefore, investigating the relationship between inflammation and depression during critical developmental stages, such as adolescence, may help to deepen our understanding of these specific biomarkers.

Although the aforementioned studies provide preliminary evidence for an inflammation–suicidality link, they are predominantly based on two-group comparisons (e.g., MDD with vs. without suicidality) and rarely focus specifically on adolescent populations. Crucially, no study has yet systematically explored CBC parameters and its derived inflammatory markers across adolescents with MDD who engage in NSSI only, SA only, or both behaviors (NSSI+SA). This distinction is clinically important, as NSSI and SA may have overlapping yet distinct biological underpinnings, and the comorbid state may represent a particularly high-risk phenotype with unique inflammatory characteristics. The failure to disaggregate these subgroups may explain prior inconsistent findings and limits our ability to develop targeted biomarker-based risk stratification tools for adolescents.

To address this gap, we investigated CBC parameters and its derived inflammatory markers in a well-characterized sample of adolescents with MDD, categorized into four clinically relevant subgroups: pure MDD (without NSSI or SA), NSSI-only, SA-only, and NSSI+SA comorbid. Specifically, we aimed to: (1) investigate potential sex-specific differences in CBC parameters and its derived inflammatory indices among adolescents with MDD; (2) examine differences in these inflammatory markers across the four clinical subgroups; and (3) evaluate the potential utility of these markers for differentiating high-risk individuals. We hypothesized that: (a) the NSSI+SA comorbid group would exhibit the most pronounced inflammatory profile, reflecting a heightened immune activation state; (b) the NSSI-only and SA-only groups might display distinct inflammatory patterns, suggesting partially divergent biological pathways; and (c) NLR would emerge as a particularly robust marker for identifying the comorbid phenotype. By systematically examining these subgroups, this study aims to provide novel insights into the biological heterogeneity underlying NSSI and SA in adolescent MDD and to identify clinically accessible biomarkers that could enhance risk assessment and guide targeted interventions.

## 2 Patients and methods

### 2.1 Ethics approval and consent to participate

This retrospective study was approved by the Ethics Committee of Hebei General Hospital (Approval No. (2024) Research Ethics Review No. (292)). The requirement for informed consent was waived due to the retrospective nature of the study.

### 2.2 Patients

This cross-sectional study was conducted in Hebei General Hospital. We retrospectively analyzed data from adolescent patients (aged 13–18 years) diagnosed with MDD and admitted to our hospital between September 2016 and May 2024.

The inclusion criteria were as follows: (1) age between 13 and 18 years; (2) diagnosis of MDD in the acute phase, according to the International Statistical Classification of Diseases and Related Health Problems, 10th Revision (ICD-10) criteria; (3) either first-episode or medication-naïve for at least two weeks prior to admission. The exclusion criteria were: (1) comorbid with other major psychiatric disorders; (2) history of smoking, drinking or substance abuse; (3) conditions potentially influencing inflammatory markers (acute infections, hematological/autoimmune diseases); (4) use of anti-inflammatory medications (e.g., corticosteroids, nonsteroidal anti-inflammatory drugs, or immunosuppressants) within one month prior to admission; (5) incomplete medical records or laboratory data.

### 2.3 Data collection

Demographic information, clinical features, and CBC data were collected for all participants. Demographic data included age, sex, and body mass index (BMI). Clinical assessment focused on the presence of NSSI and SA. All data were retrieved from electronic medical records.

A history of NSSI and/or SA was determined through a comprehensive review of medical records. Patients were required to have documented at least one episode of NSSI and/or SA within the past year to be identified as positive for these behaviors. Based on this classification, patients were categorized into four distinct clinical subgroups: the pure MDD group (without NSSI and SA), the NSSI-only group, the SA-only group, and the NSSI+SA comorbid group.

Fasting blood samples were collected from all patients between 6:00 a.m. and 8:00 a.m. on the second day of hospitalization. CBC was performed using a Sysmex XN-10 automated hematology analyzer (Sysmex Corporation, Japan). The measured parameters included white blood cell count (WBC, ×10^9^/L), neutrophil count (NE, ×10^9^/L), lymphocyte count (LY, ×10^9^/L), monocyte count (MO, ×10^9^/L), platelet count (PLT, ×10^9^/L), mean platelet volume (MPV, fL), platelet crit (PCT, %), and platelet distribution width (PDW, fL).

The following inflammatory indices were derived from the CBC parameters using the formulas below:

NLR (neutrophil-to-lymphocyte ratio) = neutrophils count/lymphocytes count;

MLR (monocyte-to-lymphocyte ratio) = monocytes count/lymphocytes count;

PLR (platelet-to-lymphocyte ratio) = platelets count/lymphocytes count;

SII (systemic immune-inflammation index) = (platelets count × neutrophils count)/lymphocytes count.

### 2.4 Statistical analysis

All statistical analyses were performed using IBM SPSS Statistics (Version 26.0). Categorical variables are presented as numbers and percentages and were compared using the Chi-square test or Fisher’s exact test, as appropriate. The normality of the distribution for continuous variables was assessed using the Kolmogorov–Smirnov test. Non-normally distributed data are presented as the median and interquartile range and were compared using the Mann–Whitney U test (for two-group comparisons) or the Kruskal-Wallis H test (for multi-group comparisons). For significant Kruskal-Wallis results, *post-hoc* pairwise comparisons used the Mann-Whitney U test with Bonferroni correction (adjusted significance level α = 0.05/6 = 0.008). Multivariate logistic regression identified variables independently associated with NSSI or SA. Receiver operating characteristic (ROC) curve analysis evaluated the diagnostic performance of significant markers, with the area under the curve (AUC) indicating predictive ability. A two-tailed p < 0.05 was considered statistically significant, except for *post-hoc* tests (p < 0.008).

## 3 Results

### 3.1 Comparison of hematological indices between sexes

A total of 618 patients were enrolled in this study, comprising 441 (71.4%) females and 177 (28.6%) males. A significant age difference was observed between sexes, with male patients being slightly older than females (*p* = 0.012) (**Table 1**). Given this age difference, subsequent multivariate analyses examining sex differences were adjusted for age.

**Table 1:** Blood cell count parameters and derived inflammatory indices: a comparison between the different sexes.

	Total sample	Male	Female	Z	*P*
n(%)	618	177(28.6%)	441(71.4%)		
Age	15(14,16)	16(14,17)	15(14,16)	-2.5	**0.012**
BMI	21.09(18.78,23.94)	21.48(18.62,25.10)	20.96(18.9,23.63)	-1.184	0.236
WBC	5.97(5.18,6.95)	6.16(5.27,7.15)	5.88(5.16,6.90)	-1.121	0.262
NE	3.04(2.40,3.77)	3.21(2.50,3.82)	2.98(2.38,3.73)	-1.31	0.190
LY	2.36(2.02,2.79)	2.29(2.01,2.79)	2.39(2.02,2.78)	-0.633	0.527
MO	0.33(0.27,0.39)	0.33(0.28,0.40)	0.32(0.26,0.38)	-1.183	0.070
PLT	270(236,311)	261(230,301)	274(240,315)	-2.677	**0.007**
MPV	10.2(9.7,10.8)	10.3(9.7,10.8)	10.2(9.7,10.8)	-0.381	0.703
PCT	0.27(0.24,0.31)	0.27(0.24,0.30)	0.28(0.25,0.32)	-2.756	**0.006**
PDW	11.5(10.3,12.6)	11.6(10.5,12.7)	11.5(10.2,12.6)	-1.364	0.172
NLR	1.26(0.97,1.64)	1.32(1.03,1.66)	1.24(0.96,1.63)	-1.233	0.218
MLR	0.13(0.11,0.16)	0.14(0.11,0.17)	0.13(0.11,0.16)	-1.771	0.077
PLR	114.62(94.19,137.44)	112.29(91.25,134.51)	116.26(96.08,138.76)	-1.508	0.132
SII	340.42(259.28,460.95)	340.49(263.55,453.02)	340.35(257.25,465.13)	-0.054	0.957

BMI, body mass index; WBC, white blood cell count; NE, neutrophil count; LY, lymphocyte count; MO, monocyte count; PLT, platelet count; MPV, mean platelet volume; PCT, platelet crit; PDW, platelet distribution width; NLR, neutrophil-to-lymphocyte ratio; MLR, monocyte-to-lymphocyte ratio; PLR, platelet-to-lymphocyte ratio; SII, systemic immune-inflammation index.

Bold values indicate statistical significance (p < 0.05)

Regarding inflammatory markers, female patients had significantly higher PLT (*p* = 0.007) and PCT (*p* = 0.006) compared to males. A trend toward significance was also observed for MLR (*p* = 0.077) and MO (*p* = 0.070), with females showing slightly lower values. No significant sex differences were found for other inflammatory markers, including NLR, PLR, or SII (all *p* > 0.05). The detailed results are presented in **Table 1**.

To further examine the independent correlates of female sex, we performed a binary logistic regression analysis with sex as the dependent variable, adjusting for age. As presented in **Table 2**, younger age (OR = 0.885, 95% confidence interval [CI]: 0.793–0.987, *p* = 0.028) and higher platelet count (OR = 1.003, 95% CI: 1.001–1.006, *p* = 0.045) were independently associated with female sex.

**Table 2:** Logistic regression analyses of clinical subgroup.

Groups	Variables	B	SE	*P*	OR (95%CI)
Male vs Female	Age PLT	-0.123 0.003	0.056 0.002	**0.028** **0.045**	0.885(0.793-0.987) 1.003(1.001-1.006)
Pure MDD vs NSSI+SA	NLR	1.068	0.285	**<0.001**	2.911(1.664-5.092)
NSSI-only vs NSSI+SA	NLR	1.305	0.355	**<0.001**	3.687(1.838-7.396)
SA-only vs NSSI+SA	NLR	1.257	0.372	**0.001**	3.514(1.693-7.291)

Pure MDD, the pure major depressive disorder group; NSSI-Only, the non-suicidal self-injury only group; SA-Only, the suicide attempts only group; NSSI+SA, the non-suicidal self-injury and suicide attempts comorbid group; PLT, platelet count; NLR, neutrophil-to-lymphocyte ratio.

Bold values indicate statistical significance (p < 0.05)

### 3.2 Comparison among clinical subgroups

#### 3.2.1 Sample characteristics and co-occurrence rates

The 618 patients were categorized into four clinical subgroups: the NSSI-only group (n=131, 21.2%), the SA-only group (n=41, 6.6%), the NSSI+SA comorbid group (n=64, 10.4%), and the pure MDD group (n=382, 61.8%) (**Table 3**). Overall, 195 patients (31.6%) engaged in NSSI, and 105 patients (17.0%) had a history of SA. Notably, among the 195 patients with NSSI, 64 (32.8%) also had a history of SA. Conversely, among the 105 patients with SA, 64 (61.0%) also engaged in NSSI. The risk of SA was significantly higher in patients with NSSI (32.8%, 64/195) than in those without NSSI (9.7%, 41/423).

**Table 3:** Comparison of blood cell count parameters and derived inflammatory indices between four clinical subgroups.

	Pure MDD	NSSI-Only	SA-Only	NSSI+SA	H(Z)/χ2	*P*	*Post-hoc*
n(%)	382(61.8%)	131(21.2%)	41(6.6%)	64(10.4%)			
Age	15(14,17)	15(14,16)	16(14,16)	15(14,16)	4.676	0.197	
Sex					26.962	<0.001	N, N+S > P
male (n, %)	137(35.9%)	21(16.0%)	10(24.4%)	9(14.1%)			
female (n, %)	245(64.1%)	110(84.0%)	31(75.6%)	55(85.9%)			
BMI	21.09(18.83, 23.94)	21.09(18.76, 23.93)	20.70(18.67, 22.68)	21.31(18.76, 24.51)	0.558	0.906	
WBC	5.89(5.02, 6.96)	5.98(5.41, 6.84)	5.47(4.88, 6.84)	6.11(5.60, 7.03)	4.671	0.198	
NE	2.99(2.37, 3.78)	3.13(2.41, 3.69)	2.64(2.24, 3.23)	3.39(2.86, 4.30)	16.515	**0.001**	N+S > P, N, S
LY	2.31(2.03, 2.76)	2.48(2.13, 2.86)	2.35(1.76, 3.17)	2.26(1.77, 2.57)	11.797	**0.008**	N > N+S
MO	0.32(0.26, 0.39)	0.33(0.28, 0.39)	0.33(0.28, 0.39)	0.34(0.28, 0.39)	1.027	0.795	
PLT	266(233, 310)	281(249, 322)	268(245, 302)	272(239,310)	6.222	0.101	
MPV	10.2(9.6, 10.8)	10.3(9.7, 10.7)	10.1(9.8, 10.8)	10.4(9.9,11.2)	4.241	0.237	
PCT	0.27(0.24,0.31)	0.29(0.26,0.33)	0.28(0.25,0.31)	0.28(0.26,0.32)	9.691	**0.021**	N > P
PDW	11.5(10.1, 12.6)	11.5(10.4, 12.3)	11.3(10.3, 12.7)	11.8(10.7,13.8)	4.819	0.186	
NLR	1.23(0.96,1.63)	1.31(0.95,1.58)	1.04(0.84,1.46)	1.50(1.17,2.38)	25.138	**<0.001**	N+S > P, N, S
MLR	0.13(0.11,0.16)	0.13(0.11,0.16)	0.13(0.10,0.18)	0.16(0.13,0.19)	10.856	**0.013**	N+S > P, N
PLR	113.79(91.51,136.32)	115.18(96.94,133.59)	108.94(90.25,140.65)	124.21(102.39,154.32)	8.937	**0.030**	N+S > P
SII	326.44(255.03,443.71)	364.99(261.53,475.79)	279.26(233.97,361.41)	433.46(295.37,671.44)	20.898	**<0.001**	N+S > P, N, S

Pure MDD, the pure major depressive disorder group; NSSI-Only, the non-suicidal self-injury only group; SA-Only, the suicide attempts only group; NSSI+SA, the non-suicidal self-injury and suicide attempts comorbid group; WBC, white blood cell count; NE, neutrophil count; LY, lymphocyte count; MO, monocyte count; PLT, platelet count; MPV, mean platelet volume; PCT, platelet crit; PDW, platelet distribution width; NLR, neutrophil-to-lymphocyte ratio; MLR, monocyte-to-lymphocyte ratio; PLR, platelet-to-lymphocyte ratio; SII, systemic immune-inflammation index; P, the pure major depressive disorder group; N, the non-suicidal self-injury only group; S, the suicide attempts only group; N+S, the non-suicidal self-injury and suicide attempts comorbid group.

Bold values indicate statistical significance (p < 0.05)

#### 3.2.2 Group differences in age and sex distribution

Age: No significant differences in age were observed across the four clinical subgroups (*p* > 0.05) (**Table 3**), suggesting that age was unlikely to confound the between-group comparisons of inflammatory markers.

Sex: A significant difference in sex distribution was observed across the four groups (H = 26.962, *p* < 0.001). *Post-hoc* pairwise comparisons with Bonferroni correction revealed that the proportion of females was significantly higher in both the NSSI-only group and the NSSI+SA group than in the pure MDD group. No significant difference was found between the SA-only group and the pure MDD group. Furthermore, sex distributions among the NSSI-only, SA-only, and NSSI+SA groups did not differ significantly. Detailed results are presented in **Table 3**.

A supplementary analysis of sex-specific progression from NSSI to SA showed that 33.3% (55/165) of females and 30.0% (9/30) of males with NSSI had a co-occurring SA, indicating a similar risk profile across sexes.

#### 3.2.3 Group differences in hematological and inflammatory indices

The Kruskal–Wallis H test indicated significant differences among the groups for neutrophil count (NE), lymphocyte count (LY), PCT, NLR, MLR, PLR, and SII (all *p* < 0.05). After Bonferroni correction, *post-hoc* comparisons yielded the following key findings. The NSSI-only group exhibited significantly higher levels of PCT than the pure MDD group. The NSSI+SA group demonstrated a distinct inflammatory profile, characterized by significantly higher levels of NE, NLR, MLR, PLR, and SII, alongside lower levels of LY, than the pure MDD group. When compared to the NSSI-only group, the NSSI+SA group had significantly higher levels of NE, NLR, MLR, and SII, and significantly lower levels of LY. Furthermore, the NSSI+SA group showed significantly higher levels of NE, NLR, and SII than the SA-only group. No significant differences in any hematological parameters were found between the SA-only group and either the pure MDD group or the NSSI-only group. Detailed results are presented in **Table 3**.

### 3.3 Multivariate logistic regression analysis of clinical subgroups

NLR as a predictor of NSSI+SA comorbidity: A higher NLR was a robust risk factor for belonging to the NSSI+SA group (**Table 2**). Individuals in this group had a significantly higher NLR than those in the pure MDD group (OR = 2.91, 95% confidence interval [CI]: 1.66-5.09, *p* < 0.001). Furthermore, the NSSI+SA group was distinguished from the other symptomatic groups, showing a higher risk than both the NSSI-only group (OR = 3.69, 95% CI: 1.84–7.40, *p* < 0.001) and the SA-only group (OR = 3.51, 95% CI: 1.69–7.29, *p* = 0.001).

Age and PLT as a predictor of female sex: In the sex regression model, younger age was independently associated with female sex (OR = 0.885, 95% CI: 0.793–0.987, *p* = 0.028). After controlling for age, higher PLT remained a slight but statistically significant risk factor for females (OR = 1.003, 95% CI: 1.001–1.006, *p* = 0.045) (**Table 2**).

### 3.4 Diagnostic performance of NLR for identifying the NSSI+SA comorbid group: an ROC curve analysis

**Figure 1:**
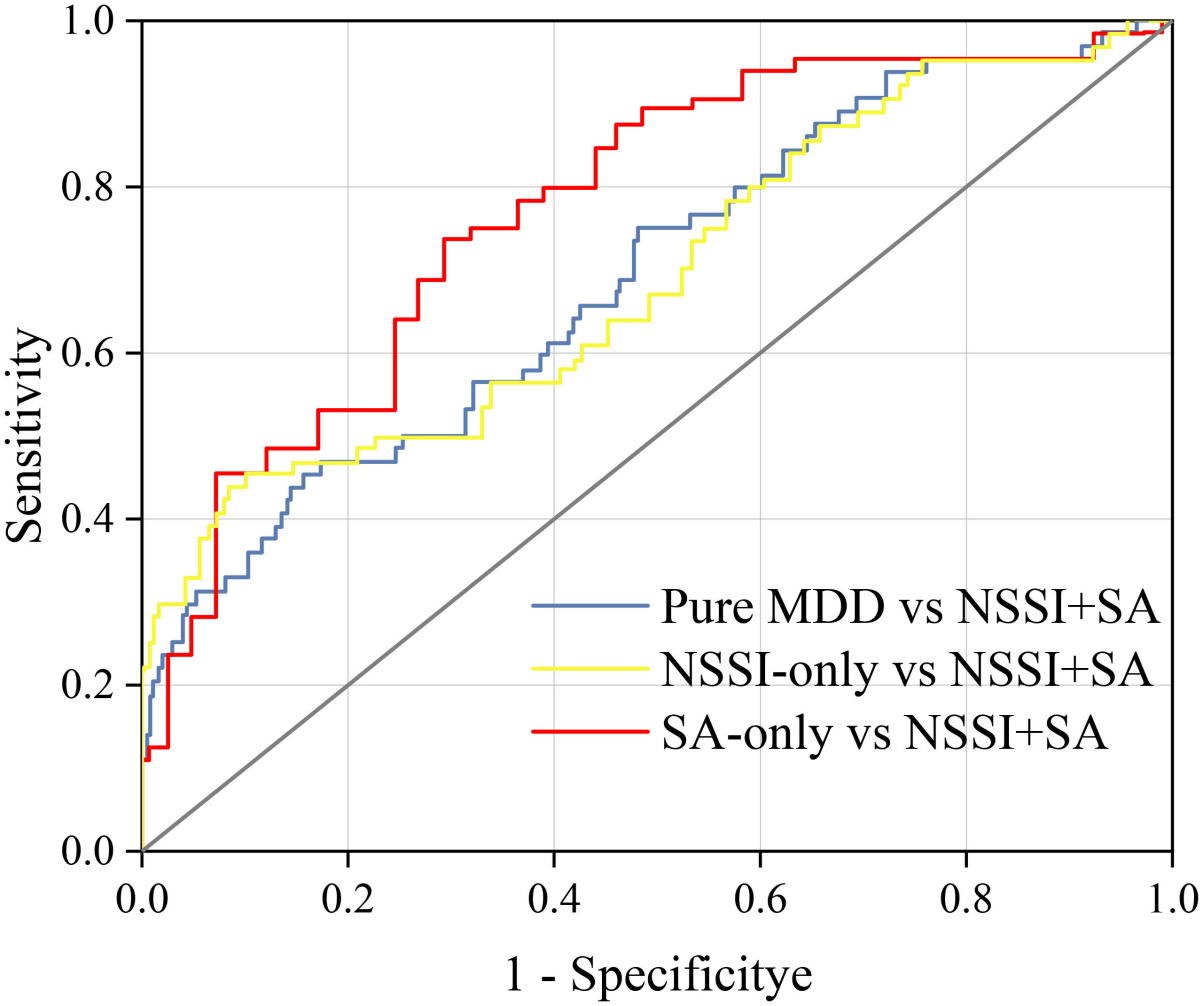
The ROC curves of NLR to differentiate the NSSI +SA comorbid group from other groups. The blue line presents differentiation of the NSSI +SA comorbid group from the pure MDD group, with an AUC of 67.4%, a sensitivity of 43.80%, and a specificity of 84.30%. The yellow line presents differentiation of the NSSI +SA comorbid group from the NSSI - only group, with an AUC of 67.4%, a sensitivity of 43.80%, and a specificity of 90.10%. The red line presents differentiation of the NSSI +SA comorbid group from the SA - only group, with an AUC of 73.6%, a sensitivity of 73.40%, and a specificity of 68.30%.

ROC curve analysis demonstrated that NLR had moderate power (AUC = 0.674) in distinguishing the NSSI+SA group from the pure MDD group and NSSI-only group with high specificity (>84%) at cut-off values of 1.83 and 1.84. It performed best in differentiating the NSSI+SA from the SA-only group (AUC = 0.736), achieving 73.4% sensitivity at a cut-off value of 1.25 (**Table 4**; **Figure 1**).

**Table 4:** ROC curve of NLR to differentiate the NSSI + SA comorbid group from other groups.

Groups	AUC	95%CI	Cut-off	Sensitivity	Specificity	*P*
Pure MDD vs NSSI+SA	0.674	0.599-0.749	1.83	0.438	0.843	**<0.001**
NSSI-Only vs NSSI+SA	0.674	0.589-0.758	1.84	0.438	0.901	**<0.001**
SA-Only vs NSSI+SA	0.736	0.636-0.835	1.25	0.734	0.683	**<0.001**

Pure MDD, the pure major depressive disorder group; NSSI-Only, the non-suicidal self-injury only group; SA-Only, the suicide attempts only group; NSSI+SA, the non-suicidal self-injury and suicide attempts comorbid group; ROC, receiver-operating characteristic; NLR, neutrophil-to-lymphocyte ratio.

Bold values indicate statistical significance (p < 0.05)

## 4 Discussion

To our knowledge, this is the first study to systematically compare differences in CBC and derived inflammatory indices by classifying adolescent patients with MDD into four clinical subgroups (pure MDD, NSSI-only, SA-only, NSSI+SA). Our main findings reveal a graded pattern of inflammatory alterations. First, the NSSI+SA comorbid group exhibited a broad and robust inflammatory signature, characterized by elevated NE, NLR, MLR, PLR, and SII, alongside reduced LY. NLR emerged as a particularly strong independent risk factor for this high-risk phenotype. Second, the NSSI-only group showed a more restricted alteration, with elevated PCT compared to the pure MDD group. Third, the SA-only group displayed no distinct inflammatory profile. Fourth, sex-specific analyses revealed higher platelet-related parameters (PLT, PCT) in females, though other inflammatory markers did not differ by sex. Together, these findings reveal distinct inflammatory correlates across clinical subgroups, with the NSSI+SA comorbid state representing a biologically severe phenotype characterized by broad immune activation. The absence of inflammatory alterations in the SA-only subgroup further underscores the biological heterogeneity underlying NSSI and SA in adolescent MDD and highlights the potential of accessible markers for risk stratification.

### 4.1 Sex differences

In our study, we observed a higher prevalence of females among adolescents with MDD (female-to-male ratio ≈ 2.5:1) and an earlier age of onset in female patients, which aligns with prior reports (36, 37). Female patients exhibited significantly higher levels of PLT and PCT compared to males, while no sex differences were found for other inflammatory markers, including NLR, MLR, PLR, and SII. This platelet-specific sex difference may reflect underlying hormonal and neuroendocrine influences. Evidence suggests that sex differences in hypothalamic-pituitary-adrenal (HPA) axis function emerge during adolescence, with females showing greater stress-induced cortisol and ACTH responses than males (38–40). HPA axis activation can promote systemic inflammation through glucocorticoid-mediated pathways (38, 41), potentially contributing to elevated platelet parameters in females. Sex hormones also play a modulating role: testosterone generally exerts anti-inflammatory effects, while estrogen has context-dependent pro- and anti-inflammatory properties (42, 43). A recent study found sex-specific correlations between hormone levels and inflammatory markers, with estradiol positively associated with NE, NLR, and SII in females but negatively associated with NE, LY, PLT, and SII in males (44).

The observation of elevated PLT in female adolescents with MDD in our study, while representing a modest effect size, is consistent with a two-year longitudinal study that also reported higher PLT in females and a temporal association between PLT levels and depressive symptoms (45). These findings support the potential of platelet-related parameters as sex-specific correlates of inflammatory status in adolescent MDD, while the absence of sex differences in other markers (NLR, MLR, PLR, SII) indicates that the broader inflammatory signature associated with suicidality does not differ substantially between males and females.

### 4.2 Elevated PCT in the NSSI-only subgroup

We observed significantly higher PCT in the NSSI-only group compared to the pure MDD group. PCT is calculated as the product of PLT and mean platelet volume (MPV), representing the total circulating platelet volume. Although neither PLT nor MPV alone reached statistical significance, both showed elevated trends in the NSSI-only group, collectively contributing to the PCT increase. Platelets serve as the primary reservoir for peripheral serotonin, storing and releasing this neurotransmitter upon activation (46–48). Through this mechanism, peripheral platelet alterations may indirectly reflect or influence central serotonergic function, which is central to mood regulation and impulse control (49, 50), both of which are core features of NSSI. Additionally, enhanced platelet activity has been associated with increased endothelial permeability and recruitment of inflammatory cells (51), processes that could theoretically contribute to peripheral inflammation. While these proposed pathways offer plausible explanations for the observed association, it is important to emphasize that none were directly assessed in the present study. The cross-sectional design precludes causal inferences, and the elevated PCT in the NSSI-only group should be interpreted as an association requiring further investigation. Future studies measuring central serotonergic function or platelet activation markers alongside PCT could help elucidate whether this peripheral finding reflects broader neurobiological processes relevant to NSSI risk.

### 4.3 Generalized immune activation in the NSSI+SA comorbid group

A key finding of this study is the broad and robust inflammatory signature observed exclusively in the NSSI+SA comorbid group, characterized by elevated NE, NLR, MLR, PLR, and SII, alongside reduced LY. NLR emerged as a particularly strong independent risk factor for this phenotype, with odds ratios ranging from 2.91 to 3.69 compared to other subgroups. This pattern suggests a state of broad innate immune activation associated with the co-occurrence of NSSI and SA. These findings align with previous studies linking elevated NLR to increased suicide risk in MDD patients (28, 29) and extend them by delineating the distinct inflammatory phenotype associated with the co-occurrence of these behaviors, rather than with either behavior alone. Notably, the NSSI+SA group differed significantly from the NSSI-only group not only in NLR but also in NE, MLR, and SII, indicating that the comorbid state is accompanied by a more extensive inflammatory response.

Several interconnected mechanisms have been proposed in the literature to explain the association between inflammation and suicidal behaviors, including monoaminergic dysregulation, kynurenine pathway activation, and neuroinflammation (33, 38, 46–49, 52–55). These pathways involve a range of neurobiological alterations, such as disrupted serotonin metabolism (46–49), increased production of neurotoxic kynurenine metabolites (52–54), microglial activation (33, 55), and structural and functional changes in brain regions critical for impulse control and emotional regulation, including the prefrontal cortex, anterior cingulate cortex, amygdala, and hippocampus (33, 38). While these pathways are well supported by the literature and offer plausible biological contexts for interpreting our findings, it is important to emphasize that none of these mechanisms were directly assessed in the present study. Our discussion of potential pathways is therefore hypothetical and intended to generate hypotheses for future research, not to assert mechanistic conclusions based on our data. Detailed explanations involving specific brain regions, microglial activation, or molecular intermediates not measured in this study should be interpreted with caution.

An additional consideration is the potential role of sex distribution in the observed group differences. The NSSI+SA group had the highest proportion of females (85.9%), and females in our sample exhibited higher platelet-related parameters (PLT, PCT). However, the key inflammatory markers that distinguished the comorbid group, including NLR, MLR, PLR, and SII, showed no significant sex differences in univariate analyses. Given the absence of sex differences in these markers, the elevated levels observed in the NSSI+SA group are unlikely to be driven solely by its higher proportion of females.

Given the cross-sectional design, we cannot determine whether this inflammatory profile precedes, follows, or co-occurs with the emergence of suicidal behavior. We interpret these findings as demonstrating a robust association between the NSSI+SA comorbid state and broad immune activation, which warrants further investigation in longitudinal studies to clarify directionality and underlying mechanisms.

### 4.4 Absence of inflammatory signatures in the SA-only subgroup

Contrary to some previous studies reporting elevated inflammatory markers in individuals with suicidal behavior (28–32), we found no significant inflammatory marker differences between the SA-only group and either the pure MDD group or the NSSI-only group. When compared to the NSSI+SA comorbid group, the SA-only group showed significantly lower levels of NE, NLR, and SII. This divergence may stem from several factors. First, our refined classification distinguishing SA with and without NSSI may have unmasked heterogeneity obscured in previous studies that combined these phenotypes. Second, the relatively small SA-only sample (n=41) may have limited statistical power to detect modest effect sizes. Third, stringent statistical correction for multiple comparisons reduced the risk of type I error. Fourth, unmeasured confounding factors, such as medication use, illness duration, or psychiatric comorbidities, may differentially influence inflammatory profiles across subgroups. Notably, a recent meta-analysis (56) found that while NLR and MLR elevations are consistently associated with suicidal behavior, PLR showed no consistent association. This heterogeneity across studies may reflect unexamined clinical differences, such as varying rates of NSSI comorbidity across study samples. Some studies reported no significant NLR differences between MDD patients and controls (57) or among MDD patients with and without suicide attempts (58). These findings suggest that “SA-only” may represent a biologically distinct subtype from SA comorbid with NSSI, warranting confirmation in future larger studies that carefully characterize NSSI history.

### 4.5 Clinical implications of NLR cut-off values

Our findings suggest that different inflammatory markers may have utility in distinct clinical contexts, though their role as standalone diagnostic tools remains limited. NLR demonstrated the most consistent associations with the high-risk NSSI+SA phenotype. This study revealed that NLR, when distinguishing the comorbid group from pure MDD or NSSI-only groups, demonstrated high specificity (84.3% and 90.1%, respectively) but low sensitivity (43.8% in both cases) at cut-offs of 1.83 and 1.84. This finding indicates that NLR could serve as a potential “rule-in” tool to identify high-risk individuals in clinical settings. A notable finding emerged when distinguishing NSSI+SA from SA-only: NLR exhibited its best overall discriminatory power (AUC = 0.736), with a shift in performance to high sensitivity (73.4%) and moderate specificity (68.3%) at a cut-off of 1.25. The high sensitivity positions NLR in this context as an effective “broad-spectrum screening” tool.

The absence of inflammatory alterations in the SA-only group and the restriction of platelet-related changes (PCT) to the NSSI-only group underscore the heterogeneity of biological correlates across clinical presentations. These null findings suggest that not all adolescents with MDD and suicidal behaviors exhibit peripheral inflammation. Other markers showing no significant differences (WBC, MO, PLT, MPV, PDW) are unlikely to add clinical value in this context.

The interpretation of these findings should also consider the potential influences of age and sex. In our sample, age did not differ across subgroups, minimizing its confounding potential. Sex differences were limited to PLT and PCT and did not extend to the broader inflammatory signature (NLR, MLR, PLR, SII) distinguishing the comorbid group, suggesting that the elevated levels in the NSSI+SA group are not driven solely by its higher proportion of females.

In summary, while no single inflammatory marker can serve as a standalone diagnostic tool, our findings support a context-dependent approach: NLR may aid in risk stratification for NSSI+SA comorbidity, PCT may help identify the NSSI-only phenotype, and the absence of inflammatory alterations in the SA-only group highlights the need for alternative biomarkers for this subgroup. These observations should be considered preliminary and require validation in independent prospective cohorts.

### 4.6 Relationship between NSSI and SA

This study elucidates two key characteristics of the NSSI-SA relationship in adolescent MDD: a strong association coupled with relative independence. First, NSSI was confirmed as a potent risk factor for SA. Adolescents with NSSI demonstrated a more than three-fold higher risk of SA than those without NSSI (32.8% vs. 9.7%). This finding underscores that any NSSI behavior should be regarded as a critical clinical marker warranting comprehensive suicide risk assessment. Second, despite this strong association, the two behaviors also demonstrate relative independence. 39% (41/105) of the SA patients had no history of NSSI, and no significant differences in peripheral inflammatory markers were observed between the SA-only and NSSI-only groups (detailed in Section 4.4). This absence of biological distinction, despite the statistical association between the behaviors, suggests that NSSI and SA may involve partially distinct underlying pathways. Clinically, these findings imply that suicide risk screening cannot rely solely on the presence of NSSI; all adolescents with MDD should receive direct and systematic suicide risk evaluation.

Regarding sex differences, although females were more likely to engage in NSSI, the proportion with co-occurring SA was similar between sexes (females: 33.3%; males: 30.0%). This suggests that once NSSI is present, suicide risk is similarly elevated in male and female adolescents, reinforcing the need for vigilant assessment regardless of sex.

## 5 Limitations

This study has several limitations that should be considered. First, its cross-sectional design precludes causal inferences regarding the relationship between NLR and NSSI/SA comorbidity. Prospective cohort studies are needed to elucidate the causal pathways. Second, all the participants were inpatients from a single hospital, which may introduce selection bias and limit the generalizability of the findings. The relatively small sample size of the “SA-only” subgroup may have further limited the statistical power for between-group comparisons. Future multi-center studies with larger and more diverse samples are warranted to enhance the reliability and generalizability of the results. Third, the assessment of NSSI and SA behaviors relied primarily on patient self-report, which is susceptible to recall bias and social desirability bias. Future research could incorporate more objective assessment methods to mitigate these potential biases. Fourth, although strict exclusion criteria were applied, it remains challenging to fully control for all potential confounding factors, such as genetic background, MDD duration, and number of depressive episodes, and medication use. Subsequent studies should systematically collect and adjust for these variables to strengthen the robustness of the findings. Fifth, the absence of significant differences in several inflammatory markers (e.g., WBC, MO, PLT, MPV, PDW) across clinical subgroups warrants consideration. This may reflect genuinely absent associations, suggesting these markers are not involved in NSSI/SA pathophysiology. Alternatively, true but subtle differences may have gone undetected due to limited statistical power (particularly in the SA-only subgroup), stringent multiple comparison correction, or unmeasured confounding. Larger studies are needed to determine whether these null findings reflect biological reality or methodological limitations. Finally, the study sample had a higher proportion of female than male patients. Future investigations should further explore the sex-specific associations between NSSI/SA behaviors and CBC parameters and derived inflammatory markers in adolescents with MDD. Furthermore, neuroimaging should be incorporated to investigate the relationship between peripheral inflammatory markers and the structure and function of brain regions related to emotional regulation and impulse control.

## 6 Conclusions

This study demonstrates that NSSI and SA behaviors in adolescents with MDD are associated with distinct inflammatory biological signatures: selective platelet-associated activation (elevated PCT) in the NSSI-only group, broad innate immune-inflammatory activation (elevated NE, NLR, MLR, PLR, SII; reduced LY) in the NSSI+SA comorbid group, and no distinct profile in the SA-only group. NLR emerged as a strong independent correlate of NSSI+SA comorbidity, with context-dependent utility for risk stratification. Clinically, these findings underscore the heterogeneity of adolescent MDD with suicidal behaviors. NLR may serve as an accessible adjunct to identify high-risk individuals, while the absence of inflammatory alterations in the SA-only group reinforces the need for universal suicide risk screening regardless of NSSI history or marker levels. Future research should prioritize longitudinal studies, multi-center validation, neuroimaging integration, and systematic assessment of confounders such as medication and illness duration.

## Data Availability

The raw data supporting the conclusions of this article will be made available by the authors, without undue reservation.
